# Antibacterial activity of *Xenorhabdus* and *Photorhabdus* isolated from entomopathogenic nematodes against antibiotic-resistant bacteria

**DOI:** 10.1371/journal.pone.0234129

**Published:** 2020-06-05

**Authors:** Paramaporn Muangpat, Manawat Suwannaroj, Thatcha Yimthin, Chamaiporn Fukruksa, Sutthirat Sitthisak, Narisara Chantratita, Apichat Vitta, Aunchalee Thanwisai

**Affiliations:** 1 Department of Microbiology and Parasitology, Faculty of Medical Science, Naresuan University, Phitsanulok, Thailand; 2 Department of Microbiology and Immunology, Faculty of Tropical Medicine, Mahidol University, Bangkok, Thailand; 3 Centre of Excellence in Medical Biotechnology (CEMB), Faculty of Medical Science, Naresuan University, Phitsanulok, Thailand; 4 Center of Excellent for Biodiversity, Faculty of Science, Naresuan University, Phitsanulok, Thailand; University of Messina, ITALY

## Abstract

*Xenorhabdus* and *Photorhabdus*, symbiotically associated with entomopathogenic nematodes (EPNs), produce a range of antimicrobial compounds. The objective of this study is to identify *Xenorhabdus* and *Photorhabdus* and their EPNs hosts, which were isolated from soil samples from Saraburi province, and study their antibacterial activity against 15 strains of drug-resistant bacteria. Fourteen isolates (6.1%), consisting of six *Xenorhabdus* isolates and eight *Photorhabdus* isolates, were obtained from 230 soil samples. Based on the BLASTN search incorporating the phylogenetic analysis of a partial *recA* gene, all six isolates of *Xenorhabdus* were found to be identical and closely related to *X*. *stockiae*. Five isolates of *Photorhabdus* were found to be identical and closely related to *P*. *luminescens* subsp. *akhurstii*. Two isolates of *Photorhabdus* were found to be identical and closely related to *P*. *luminescens* subsp. *hainanensis*. The remaining isolate of *Photorhabdus* was found to be identical to *P*. *asymbiotica* subsp. *australis*. The bacterial extracts from *P*. *luminescens* subsp. *akhurstii* showed strong inhibition the growth of *S*. *aureus* strain PB36 (MSRA) by disk diffusion, minimal inhibitory concentration, and minimal bactericidal concentration assay. The combination between each extract from *Xenorhabdus*/*Photorhabdus* and oxacillin or vancomycin against *S*. *aureus* strain PB36 (MRSA) exhibited no interaction on checkerboard assay. Moreover, killing curve assay of *P*. *luminescens* subsp. *akhurstii* extracts against *S*. *aureus* strain PB36 exhibited a steady reduction of 10^5^ CFU/ml to 10^3^ CFU/ml within 30 min. This study demonstrates that *Xenorhabdus* and *Photorhabdus*, showed antibacterial activity. This finding may be useful for further research on antibiotic production.

## Introduction

Antibiotic-resistant bacteria have become an emerging public health problem. *Pseudomonas aeruginosa*, *Klebsiella pneumoniae*, *Escherichia coli*, *Staphylococcus aureus*, *Acinetobacter baumannii*, and *Enterococcus faecalis* are the major sources of antibiotic-resistant bacteria [1]. These bacteria develop new resistance mechanisms with the emergence and the spread of the disease. This leads to higher cost of healthcare, longer duration of illness, use of more expensive drugs, and the success of prevention and treatment. Antibiotics are used as a general antimicrobial therapy to effectively treat infections. At present, the number of antibiotics effective against drug resistance is declining, predisposing us toward a future without effective antibiotics [2–5]. It is difficult to get accurate estimates of antimicrobial-resistant bacterial infections which are predicted lead to nearly 10 million deaths per year by 2050 [6]. Therefore, alternative treatments are needed against antibiotic-resistant bacteria. Biological compounds from natural or bacterial resources are one such alternative approach. *Xenorhabdus* and *Photorhabdus*, the symbiotic bacteria associated with entomopathogenic nematodes (EPNs), have been reported to be bacterial resources for the production of antimicrobial compounds. Their cell suspension and metabolite compound activities effectively inhibit the growth of *Staphylococcus pyogenes* and *S*. *aureus* [7,8], *Bacillus subtilis*, *Botrytis cinerea* [9], *Escherichia coli*, *Klebsiella pneumoniae*, *Enterobacter coloacae* [10], *Fusicladium effusum* [11], *Bacillus anthracis*, *Phytophthora capsici*, and *Rhizoctonia solani* [12].

*Xenorhabdus* and *Photorhabdus* are insect pathogenic Gram-negative bacilli belonging to the Enterobacteriaceae family that areintestinal symbionts of the infective juvenile (IJ) stage of nematodes in the families Steinernematidae and Heterorhabditidae, respectively. The IJ can penetrate the diverse insect hosts via natural openings, e.g., mouth, spiracle, and anus and enter to digestive tract and hemocoel of the hosts. Upon entry, the nematodes release their symbiotic bacteria into the insect hemolymph where the bacteria multiply. The presence of large numbers of the bacterial symbionts has result in death of the insect larvae within 24–48 h [13]. Within the hemocoel of insect carcass the bacteria grow to stationary phase while the nematodes develop and sexually reproduce. During the final stage of development, the bacteria are able to colonize the intestine of the next generation of the IJs, and then the IJs emerge from the insect carcass to search for a new insect host. Information regarding the association between EPNs and their symbiotic bacteria is scarcely reported in Thailand. The *Steinernema siamkayai* associated with *X*. *stockiae* was first described by Tailliez et al. [14]. Later, *H*. *indica*, in association with *P*. *luminescens*, was found in the Khon Kaen (northeastern Thailand) and Krabi (southern Thailand) provinces [15]. Diversity of association between symbiotic bacteria and EPNs has been reported: *X*. *stockiae* associated with *S*. *websteri*, *X*. *miraniensis* lived with *S*. *khoisanae*, *P*. *luminescens* lived with *H*. *indica*, *H*. *baujardi*, *H*. sp. SGgi or *H*. sp. SGmg3, *P*. *asymbiotica* lived with *H*. *indica* [16], and *Xenorhabdus* sp. associated with *S*. *websteri* and was isolated from lower northern Thailand [17].

At the present, 26 species of *Xenorhabdus* and five species of *Photorhabdus* have been documented worldwide, together with approximately 100 species of EPNs [16–23]. These bacteria produce a broad range of secondary metabolites, including antimicrobial, insecticidal, and cytotoxic activities [8]. Antimicrobial compounds include benzaldehyde [12], 1-carbapen-2-em-3-carboxylic acid [10], 3, 5-dihydroxy-4-isopropystilbene [9], 3-hydroxy-2-isopropyl-5-phenethylphenyl carbamate [24], 2-isopropyl-5-(3-phenyl-2-oxiranyl0-benzene-1,3diol [25], and chaiyaphumine [26]. In the recent year, antibiotic-resistant bacteria are emerging with global spread leads to difficulties for the control of the disease. We expected that the crude extracts of *Xenorhabdus* or *Photorhabdus* combination with antibiotics could inhibit the growth of antibiotic-resistant bacteria. Natural products have been an unlimited source of biologically- active compound. Although several compounds have emerged from the process of drug discovery, many steps are needed to further carry on to achieve the goals and reduce the burden of antimicrobial-resistant bacteria. The US food and drug administration (FDA) has classified the main processes for drug development as follows: (1) discovery and development in the laboratory, (2) preclinical research in the laboratory and animal testing of the drugs, (3) clinical research in human for safety and efficacy, (4) FDA review for approval of data related to the drugs, and (5) FDA post-market safety monitoring when products are usable for the public [27].

Despite technological advances in pharmaceutical productions, there is still a need to identify new potential antibiotics against the antibiotic-resistant bacteria from different resources. The objective of this study was to study the antibacterial activities of *Xenorhabdus* and *Photorhabdus* against antibiotic-resistant bacteria. *Xenorhabdus* and *Photorhabdus*, associated with the EPNs collected from Saraburi province, were identified using molecular techniques. In addition, a phylogenetic tree of *Xenorhabdus* and *Photorhabdus* was constructed to determine their phylogenetic relationship.

## Materials and methods

### Soil collection and EPNs isolation

The Saraburi province in central Thailand was selected as the soil sampling site. Samples were taken from a diverse of habitats, for example, natural grassland, roadside verges, woodland, and bank of ponds and rivers. No specific permission was required for the collection of soil samples. For each site, 5 soil samples were randomly taken in an area of approximately 100 m^2^ at a depth of 10–20 cm using a hand shovel. Approximately 500 g of each soil sample was placed into a plastic bag [16]. A total of 46 sites were sampled, and 230 soil samples were collected from the Mueang Saraburi, Phra Phutthabat, Kaeng Khoi, and Sao Hai districts. The EPNs were isolated from the soil samples using the *Galleria mellonella* baiting technique as described by Bedding and Akhurst [28]. White traps were used to isolate the emerging infective juvenile EPNs from the *G*. *mellonella* cadavers [29]. The larval nematodes were kept at 13–15°C in distilled water prior to molecular identification.

### Identification of EPNs

PCR amplification and sequencing of a partial region of 28S rDNA gene or internal transcribed spacer (ITS) were performed according to Hominick et al. and Stock et al [30,31]. The primers TW81_F (5’-GTTTCCGTAGGTGAACCTGC-3’) and AB28_R (5’-ATATGCTTAAGTTCAGCGGGT-3’) were used to amplify a region of internal transcribed spacers for *Heterorhabditis*, while 539_F (5’GGATTTCCTTAGTAACTGCGAGTG-3’) and 535_R (5’TAGTCTTTCGCCCCTATACCCTT-3’) were used to amplify a region of 28S rDNA for *Sternernema*. The genomic DNA samples of nematodes were extracted using a protocol described previously in Thanwisai et al. [16]. The PCR conditions were as described in Thanwisai et al. and Vitta et al. [16,17]. The PCR components (30 μl) comprised of 7.5 μl of a DNA-extracted solution (approximately 200 μg), 0.6 μl of 200 μM dNTPs, 1.2 μl of 5μM each primer, 4.2 μl of 25 mM MgCl_2_, 3 μl of 10X buffer, 0.3 μl of 5 U/ml Tag polymerase, and 12 μl of distilled water. The cycling conditions for ITS were used as follows: one cycle of 95°C for 5 min, followed by 35 cycles of 94°C for 1 min, 50°C for 30 sec, 72°C for 1 min, and a final extension at 72°C for 7 min. The cycling conditions for 28s rDNA were used as follows: one cycle of 95°C for 5 min, followed by 35 cycles of 94°C for 1 min, 55°C for 30 sec, 72°C for 45 sec, and a final extension at 72°C for 7 min. Both PCR conditions were performed in the Applied Biosystems thermal cycler (Applied Biosystems^TM^ Veriti^TM^ thermal cycler, Pittsburgh, USA). The PCR products were visualized on ethidium bromide-stained agarose gel and purified using a Gel/PCR DNA Fragments Extraction Kit (Geneaid Biotech Ltd., Taiwan). Sequencing was performed by Macrogen Inc. (Korea). A BLASTN search was performed against a nucleotide database to identify EPN species (http://www.ncbi.nlm.nih.gov/blast/Blast.cgi). A similarity above 97% was considered as the same species.

### Isolation, identification, and phylogenetic tree of *Xenorhabdus* and *Photorhabdus*

*Xenorhabdus* and *Photorhabdus* were isolated from the haemolymph of dead *Galleria mellonella*, which was infected with EPNs. Approximately 1 μl of haemolymph was streaked on nutrient bromothymol blue triphenyltetrazolium chloride agar (NBTA) (HiMedia, Mumbai, India), which is a selective and differential medium. A dark green colony of *Photorhabdus* and a blue colony of *Xenorhabdus* were observed after incubation in the dark at room temperature for 3–4 days [16]. A single colony from each isolate was sub-cultured on NBTA. All the bacterial isolates were kept in a Luria Bertani broth (HiMedia, Mumbai, India) containing 20% glycerol at -40°C.

Species identification and phylogenetic analysis of *Xenorhabdus* and *Photorhabdus* isolates were performed based on a partial *recA* sequence. Genomic DNA was extracted from a 3 ml LB overnight culture of *Xenorhabdus* and *Photorhabdus* using a Genomic DNA Mini Kit (Geneaid Biotech Ltd., Taiwan). A set of primers, recA_F (5′-GCTATTGATGAAAATAAACA-3′) and recA_R (5′- RATTTTRTCWCCRTTRTAGCT-3′), was used to amplify an 890 bp region of the *recA* gene [32]. A total volume of 30 μl PCR reagent contained 3 μl of DNA extract, 0.6 μl of 200 μM dNTPs, 1.2 μl of 5μM of each primer, 4.2 μl of 25mM MgCl_2_, 3 μl of 10X reaction buffer, 0.3 μl of 5U/ml Tag polymerase, and 12 μl of distilled water, as carried out in the Applied Biosystems thermal cycler (Applied Biosystems^TM^ Veriti^TM^ thermal cycler, Pittsburgh, USA) with PCR parameters and as described by Thanwisai et al. [16]. PCR products were visualized on ethidium bromide-stained agarose-gel electrophoresis and purified using a Gel/PCR DNA Fragment Extraction Kit (Geneaid Biotech Ltd., Taiwan). The purified PCR products were sequenced by Macrogen Inc. To identify *Xenorhabdus*/*Photorhabdus* into species level, BLASTN search against the NCBI nucleotide database (http://www.ncbi.nlm.nih.gov/blast/Blast.cgi) was performed using a partial *recA* gene. Multiple nucleotide sequences representing all the known species and subspecies of *Xenorhabdus* and *Photorhabdus* were downloaded from the NCBI database, aligned with the sequences from the study isolates, and trimmed to 588 bp using Clustal W included in MEGA software version 7.0. A phylogenetic tree was constructed by maximum likelihood (ML) and neighbor joining (NJ) with 1,000 bootstrap replicates using the Nearest-Neighbor-Interchange (NNI) and Tamura-Nei model by MEGA software version 7.0. Bayesian analysis was performed based on the Markov chain Monte Carlo method in MrBayes v3.2. The *recA* sequences were deposited in Genbank under the accession number MK478066 to MK478071 for six *Xenorhabdus* isolates and MK478072 to MK478079 for *eight Photorhabdus* isolates.

### Antibacterial activity of *Xenorhabdus* and *Photorhabdus* against antibiotic-resistant bacteria

#### Preparation of antibiotic-resistant bacteria

The experiments involving antibiotic-resistant bacteria were approved by Naresuan University Institutional Biosafety Committee (NUIBC MI62-06-25). Fifteen strains of antibiotic-resistant bacteria, including *Acinetobacter baumannii* (four clinical strains), *Escherichia coli* (two clinical strains), *E*. *coli* ATCC35218, *Klebsiella pneumoniae* (two clinical strains), *K*. *pneumoniae* ATCC700603, *Enterococcus faecalis* ATCC51299, *Pseudomonas aeruginosa*, *Staphylococcus aureus* (two clinical strains), and *S*. *aureus* ATCC20475 were used as pathogens for antibacterial activity. These bacteria were streaked on Mueller–Hinton agar (MHA) and incubated at 37°C for 24 h. A single colony was dissolved in 0.85% sodium chloride (NaCl), and the turbidity was adjusted to 0.5 McFarland standards. One hundred microliters of the bacterial suspension was swabbed on MHA for disk diffusion method [33].

#### Screening of *Xenorhabdus* and *Photorhabdus* isolates

To initially evaluate the antibacterial activity of whole cell culture of *Xenorhabdus* and *Photorhabdus* bacteria against antibiotic resistant bacteria, the *Xenorhabdus* and *Photorhabdus* isolates were cultured on NBTA for four days at room temperature. A single colony from each isolate was transferred into LB broth and cultured with shaking at room temperature for 48 h. The whole cell suspension of *Xenorhabdus* and *Photorhabdus* was used for the screening of antibacterial activity. Twenty μl of whole cell suspension was dropped on a Mueller–Hinton (MH) agar plated with antibiotic-resistant bacteria. The plates were then incubated at 37°C for 24 h. A clear zone from the edge of growth colony of *Xenorhabdus* and *Photorhabdus* was read as positive. The *Xenorhabdus* and *Photorhabdus* isolates that showed potential inhibition of at least one antibiotic-resistant bacteria were selected for metabolic extraction in the disc sensitivity test.

#### Bacterial extracts

A single colony of *Xenorhabdus* and *Photorhabdus* on NBTA was transferred and cultured in a 1000 ml flask containing 500 ml LB. The culture was incubated at room temperature with shaking at 180 rpm for 72 h. For extraction, 1000 ml of ethyl acetate was added to the culture and mixed well. The flask was then allowed to stand at room temperature for 24 h. All bacterial extracts were concentrated using a rotary vacuum evaporator (Buchi, Flawil, Switzerland). The extraction was performed thrice to maximize the level of crude compounds. The extract was dried under laminar airflow and stored at -20°C until it was used for the disk diffusion method.

#### Disk diffusion method

Bacterial extracts from *Xenorhabdus* and *Photorhabdus* were dissolved in dimethyl sulfoxide (*DMSO*) to a concentration 500 mg/ml. Ten microliters of the solution were dropped into a sterile 6 mm paper disc, which was then placed on the MHA plated with antibiotic-resistant bacteria. Antibiotic discs with vancomycin, tigecycline, ampicillin, ceftazidime, and ceftazidime/clavulanic acid were used as positive control and disc with DMSO was used as a negative control. The plates were then incubated at 37°C for 24 h. The diameter of clear zone (millimeter), representing the zone of inhibition, was measured in millimeter using a ruler.

#### Minimum Inhibitory Concentrations (MIC) and Minimal Bactericidal Concentrations (MBC)

Bacterial extracts with the most effective result in disk diffusion method were further evaluated by minimum inhibitory concentrations (MIC) using broth microdilution method. Two-fold serial dilutions of bacteria extract were performed in a 96-well micro titer plate. Antibiotic-resistant bacteria cultured, antibiotic-resistant bacteria cultured mixed with DMSO, and sterile Mueller-Hinton (MH) broth were used as control. Plates were incubated at 37°C for 24 h. The MIC is defined as the lowest concentration of extract in which there is no visible growth of testing bacteria in the well. In addition, the minimum bactericidal concentrations (MBC) was evaluated. Ten microliters from each well of 96-well micro titer plates from MIC was sub-cultured onto the MHA plates. The plates were then incubated at 37°C for 24 h. The lowest concentration of each extract without growth of bacteria was considered as MBC.

#### Checkerboard determination of synergistic effect of combined drugs

Bacterial extracts exhibiting highest MIC and MBC against *S*. *aureus* strain PB36 (clinical isolate) were further evaluated using Checkerboard assay. Antimicrobial combinations were performed following Teethaisong et al. [34]. The methods of bacterial culture and preparation of antibacterial agents were performed as described for the MIC broth microdilution. Fifty μl of Cation–Mueller–Hinton broth (CaMHB) was transferred into each well of 96-well micro titer plate. The antibiotics (vancomycin and oxacillin) of the combination were two-fold serially diluted along the ordinate, while the bacterial extracts were diluted along the abscissa. Each well was inoculated with 100 μl of a *S*. *aureus* strain PB36 suspension (0.5 MacFarland). The plates were incubated at 37°C for 24 h. The MICs were determined as the lowest concentration drugs in combination with bacterial extracts. The fractional inhibitory concentration (FIC) index or FICI was calculated as follows: FIC index = FIC A + FIC B, where FIC A is the MIC of drug A in the combination/MIC of drug A alone, and FIC B is the MIC of drug B in the combination/MIC of drug B alone. The combination is considered synergistic when FICI ≤ 0.5, no interaction when the FICI is 0.5–4.0, and antagonism when the FICI is > 4.0.

#### Time killing assay

The bacterial extract at 1× MIC, was mixed with a culture of *S*. *aureus* strain PB36 (MRSA) in the CaMHB and then adjusted to a final inoculum of 10^5^ CFU/ml. The mixture was diluted and counted using a drop plate technique on MHA at following time point 0, 30 min, 1h, 2h, 3h 4h, 5h, 6 h and 24 h. The plate was then incubated at 37°C. The growing colonies were counted after 24 h. The variability of *S*. *aureus* strain PB36 (MRSA) treated with the bacterial extracts at different time was statistically analyzed by one-way analysis of variance (ANOVA) followed by multiple comparison, using the Bonferroni correction (STATA version 13). The *P*-value lower than 0.05 was considered as significant difference.

## Results

### EPNs isolation and identification

A total of 46 sample sites with 230 soil samples yielded 14 isolates of EPNs belonging to the genus *Steinernema* (six isolates) and *Heterorhabditis* (eight isolates). After the BLASTN search, six isolates of *Steinernema* were identified as *S*. *surkhetense* (99% identity) while two isolates of *Heterorhabditis* were identified as *H*. *indica* (99% identity). The remaining six isolates of *Heterorhabditis* were unidentified at the species level because of the low amount of genomic DNA.

### *Xenorhabdus* and *Photorhabdus* isolation and identification

Six isolates of *Xenorhabdus* and eight isolates of *Photorhabdus* were isolated from the 14 EPNs isolates. The colony of *Xenorhabdus* was blue on NBTA agar, while *Photorhabdus* was green. Based on a region of the *recA* gene, *Xenorhabdus* was identified as *X*. *stockiae* (six isolates with 98–99% identity), while *Photorhabdus* was identified as *Photorhabdus luminescens* subsp. *akhurstii* (five isolates with 98% identity), *Photorhabdus luminescens* subsp. *hainanensis* (two isolates with 98–99%), and *Photorhabdus asymbiotica* subsp. *australis* (one isolate with 100% identity).

### Phylogenetic tree of *Xenorhabdus* and *Photorhabdus*

**Fig 1:**
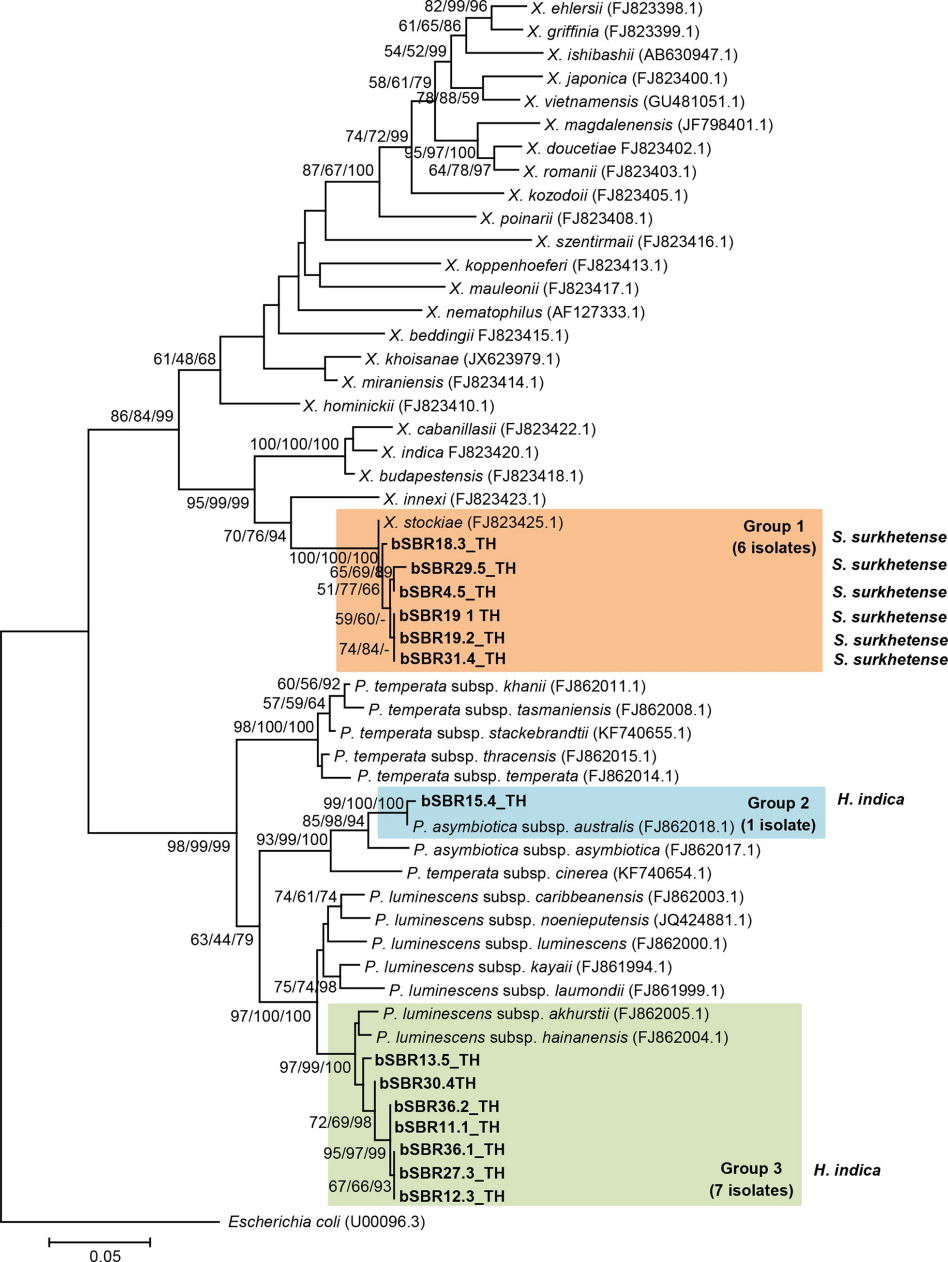
Maximum likelihood tree based on a 588 bp region of *recA* for six *Xenorhabdus* isolates and eight *Photorhabdus* isolates from Saraburi province, Thailand (codes ending with TH), together with the *Xenorhabdus* and *Photorhabdus* sequences downloaded from GenBank. The bootstrap values are based on 1,000 replicates. The numbers shown above the branches are bootstrap percentages of Maximum likelihood, Neighbor-joining and Bayesian posterior probabilities, respectively) for clades, supported above the 50% level. The bar indicates 1% sequence divergence. The EPN species from which they were isolated are also shown.

Maximum likelihood (ML) tree of *Xenorhabdus* revealed that six isolates of *Xenorhabdus* in the present study were clustered with *X*. *stockiae* strain TH01. The maximum likelihood tree of seven isolates of *Photorhabdus* was grouped with *Photorhabdus luminescens* subsp. *hainanensis* strain C8404 and *Photorhabdus luminescens* subsp. *akhurstii* strain FRG04, while one isolate of *Photorhabdus* was placed in the group of *Photorhabdus asymbiotica* subsp. *australis* strain 9802892 (**Fig 1**).

### Antibacterial activity

**Fig 2:**
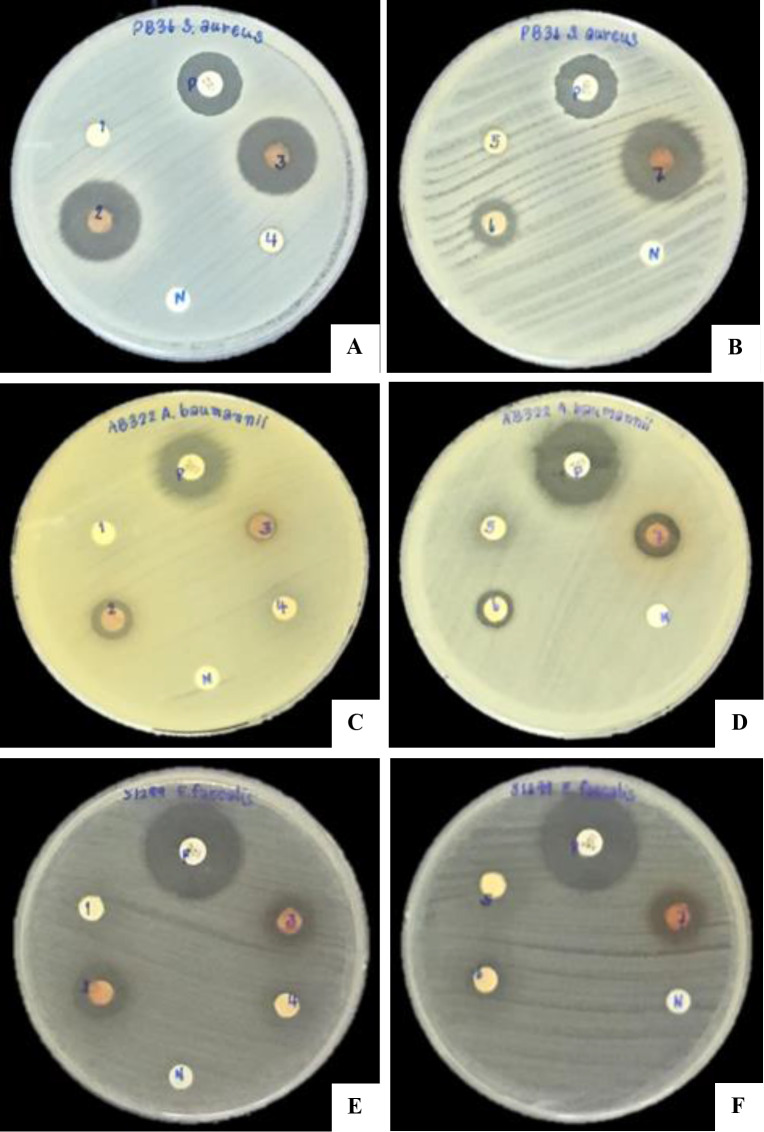
Disk diffusion method of bacterial extracts against three antibiotic-resistant bacteria. Clear zone of *S*. *aureus* strain PB36 (MRSA; A and B), *A*. *baumannii* strain AB322 (MDR; C and D), *E*. *faecalis* ATCC51299 (E and F) after exposure to bacterial extracts from bSBR4.5_TH *X*. *stockiae* (1) bSBR11.1_TH *P*. *luminescens* subsp. *akhurstii* (2), bSBR12.3_TH *P*. *luminescens* subsp. *akhurstii* (3), bSBR18.3_TH *X*. *stockiae* (4), bSBR19.1_TH *X*. *stockiae* (5), bSBR31.4_TH *X*. *stockiae* (6), bSBR36.2_TH *P*. *luminescens* subsp. *akhurstii* (7), antibiotic disks (P) and negative control (N).

Screening of whole cell culture and the extracts by the disk diffusion method demonstrated that seven isolates of *Xenorhabdus* and *Photorhabdus* showed the potential inhibition of the growth of antibiotic-resistant bacteria **(Table 1 and Fig 2)**. Four bacterial extracts, including *X*. *stockiae* (bSBR31.4_TH) and *P*. *luminescens* subsp. *akhurstii* stains bSBR11.1_TH, bSBR12.3_TH, and bSBR36.2_TH could inhibit *S*. *aureus* ATCC20475, *S*. *aureus* strain PB36 (MRSA), *S*. *aureus* strain PB57 (MRSA), *A*. *baumannii* strain AB320 (extensively drug resistant or XDR), *A*. *baumannii* strain AB321 (multi drug resistant or MDR), *A*. *baumannii* strain AB322 (MDR), and *E*. *faecalis* ATCC51299. In contrast, three bacterial extracts from *X*. *stockiae* stains bSBR4.5_TH, bSBR18.3_TH, and bSBR19.1_TH were unable to inhibit any antibiotic-resistant bacteria by the disk diffusion method. *P*. *luminescens* subsp. *akhurstii* bSBR36.2_TH showed the most board–range inhibition of up to 11 strains of antibiotic-resistant bacteria. These included *A*. *baumannii* strain AB320 (XDR), *A*. *baumannii* strain AB321 (MDR), *A*. *baumannii* strain AB322 (MDR), *A*. *baumannii* strain AB324 (XDR), *S*. *aureus* ATCC20475, *S*. *aureus* strain PB36 (methicillin-resistant *S*. *aureus* or MRSA), *S*. *aureus* strain PB57 (MRSA), *E*. *coli* strain PB1 (Extended Spectrum Beta- Lactamase or ESBL and MDR), *E*. *coli* strain PB231 (ESBL and Carbapenem-resistant Enterobacteriaceae or CRE), *P*. *aeruginosa* strain PB30 (MDR), and *E*. *faecalis* ATCC51299.

**Table 1:** Antibacterial activity of *Xenorhabdus* and *Photorhabdus* extracts against antibiotic-resistant bacteria as assessed by disk diffusion.

Bacteria (code)	Inhibit the growth of drug-resistant bacteria*														
	*A*. *baumannii* strain AB320 (XDR) ^a^	*A*. *baumannii* strain AB321 (MDR) ^b^	*A*. *baumannii* strain AB322 (MDR) ^b^	*A*. *baumannii* strain AB324 (XDR) ^a^	*S*. *aureus* ATCC20475	*S*. *aureus* strain PB36 (MRSA)^c^	*S*. *aureus* strain PB57 (MRSA)^c^	*E*. *coli* ATCC35218	*E*. *coli* strain PB1 (ESBL and MDR) ^d^^,^^b^	*E*. *coli* strain PB231 (ESBL and CRE) ^d^^,^^e^	*P*. *aeruginosa* strain PB30 (MDR)^b^	*E*. *faecalis* ATCC51299	*K*. *pneumonia* ATCC700603	*K*. *pneumoniae* strain PB5 (ESBL and MDR) ^d^^,^^b^	*K*. *pneumoniae* strain PB21 (ESBL and CRE) ^d^^,^^e^
*X*. *stockiae* (bSBR4.5_TH)	-	-	-	-	-	-	-	-	-	-	-	-	-	-	-
*P*. *luminescens* subsp. *akhurstii* (bSBR11.1_TH)	+	+	+	+	+++	+++	+++	-	+	-	-	++	-	-	+
*P*. *luminescens* subsp. *akhurstii* (bSBR12.3_TH)	+	+	+	-	+++	+++	++	-	-	-	-	++	-	-	-
*X*. *stockiae* (bSBR18.3_TH)	-	-	-	-	-	-	-	-	-	-	-	-	-	-	-
*X*. *stockiae* (bSBR19.1_TH)	-	-	-	-	-	-	-	-	-	-	-	-	-	-	-
*X*. *stockiae* (bSBR31.4_TH)	+	+	+	-	++	++	+	-	-	-	-	+	-	-	-
*P*. *luminescens* subsp. *akhurstii* (bSBR36.2_TH)	+	+	++	+	+++	+++	++	-	+	+	+	++	-	-	-

*- No activity (6 mm), + weak inhibition (7–10 mm.), ++ moderate/average inhibition (11–15 mm.), +++ stronger inhibition (16–20 mm.)

^a^ Extensively drug resistant

^b^ multidrug resistance

^c^ methicillin resistance *Staphylococcus aureus*, and

^d^ extended spectrum beta-lactamase

^e^Carbapenem-resistant Enterobacteriaceae

Based on the result of MIC and MBC, one isolate of *X*. *stockiae* and three isolates of *P*. *luminescens* subsp. *akhurstii* were further evaluated against five strains of antibiotic-resistant bacteria, including *S*. *aureus* strain PB36 (MRSA), *S*. *aureus* strain PB57 (MRSA), *A*. *baumannii* strain AB321 (MDR), *A*. *baumannii* strain AB322 (MDR), and *E*. *faecalis* ATCC51299. The inhibitory effect of bacterial extracts on these antibiotic-resistant bacteria showed the MICs and MBCs ranging from 7.81 to 0.98 mg/ml **(Table 2)**.

**Table 2:** Antibacterial activity of *Xenorhabdus* and *Photorhabdus* extracts against antibiotic-resistant bacteria as assessed by minimum inhibitory concentrations and minimal bactericidal concentration.

Bacteria (code)	Concentration of inhibition (mg/ml)									
	*S*. *aureus* strain PB36 (MRSA)		*S*. *aureus* strain PB57(MRSA)		*A*. *baumannii* strain AB321 (MDR)		*A*. *baumannii* strain AB322 (MDR)		*E*. *faecalis* ATCC51299	
	MIC	MBC	MIC	MBC	MIC	MBC	MIC	MBC	MIC	MBC
*P*. *luminescens* subsp. *akhurstii* (bSBR11.1_TH)	0.98	0.98	0.98	0.98	0.98	0.98	1.95	3.90	3.90	7.81
*P*. *luminescens* subsp. *akhurstii* (bSBR12.3_TH)	0.98	0.98	0.98	0.98	ND	ND	3.90	7.81	3.90	7.81
*X*. *stockiae* (bSBR31.4_TH)	3.90	7.81	ND	ND	ND	ND	7.81	7.81	ND	ND
*P*. *luminescens* subsp. *akhurstii* (bSBR36.2_TH)	0.98	0.98	ND	ND	ND	ND	3.90	3.90	1.95	3.90

ND = Not determined

In the checkerboard assay, the results of the combination against *S*. *aureus* strain PB36 (MRSA) are shown in **Table 3**. Based on the FIC index calculation, the combination of oxacillin and bacterial extracts exhibited no interaction with FIC index 0.53 and 1. Moreover, the combination of vancomycin and bacterial extracts exhibited no interaction with FIC index at 1.

**Table 3:** MIC (mg/ml) and FIC index of Oxacillin and Vancomycin when used either alone or in combination with bacterial extracts against *S*. *aureus* strain PB36 (MRSA).

Combination of agents	MIC (mg/ml) in combination (A+ B)	FIC index*	Type of interaction
*P*. *luminescens* subsp. *akhurstii* (bSBR11.1_TH)	0.0153	1	No interaction
Oxacillin	0.156		
*P*. *luminescens* subsp. *akhurstii* (bSBR12.3_TH)	0.49	0.53	No interaction
Oxacillin	0.0049		
*X*. *stockiae* (bSBR31.4_TH)	0.06125	1	No interaction
Oxacillin	0.156		
*P*. *luminescens* subsp. *akhurstii* (bSBR36.2_TH)	0.49	0.53	No interaction
Oxacillin	0.0049		
*P*. *luminescens* subsp. *akhurstii* (bSBR11.1_TH)	0.0153	1	No interaction
Vancomycin	0.003125		
*P*. *luminescens* subsp. *akhurstii* (bSBR12.3_TH)	0.0153	1	No interaction
Vancomycin	0.003125		
*X*. *stockiae* (bSBR31.4_TH)	0.06125	1	No interaction
Vancomycin	0.003125		
*P*. *luminescens* subsp. *akhurstii* (bSBR36.2_TH)	0.0153	1	No interaction
Vancomycin	0.003125		

*FIC index = FIC A + FIC B, where FIC A is the MIC (mg/ml) of drug A in the combination/MIC of drug A alone, and FIC B is the MIC of drug B in the combination/MIC of drug B alone. The combination is considered synergistic when FICI ≤ 0.5, no interaction when the FICI is 0.5–4.0, and antagonism when the FICI is > 4.0.

**Fig 3:**
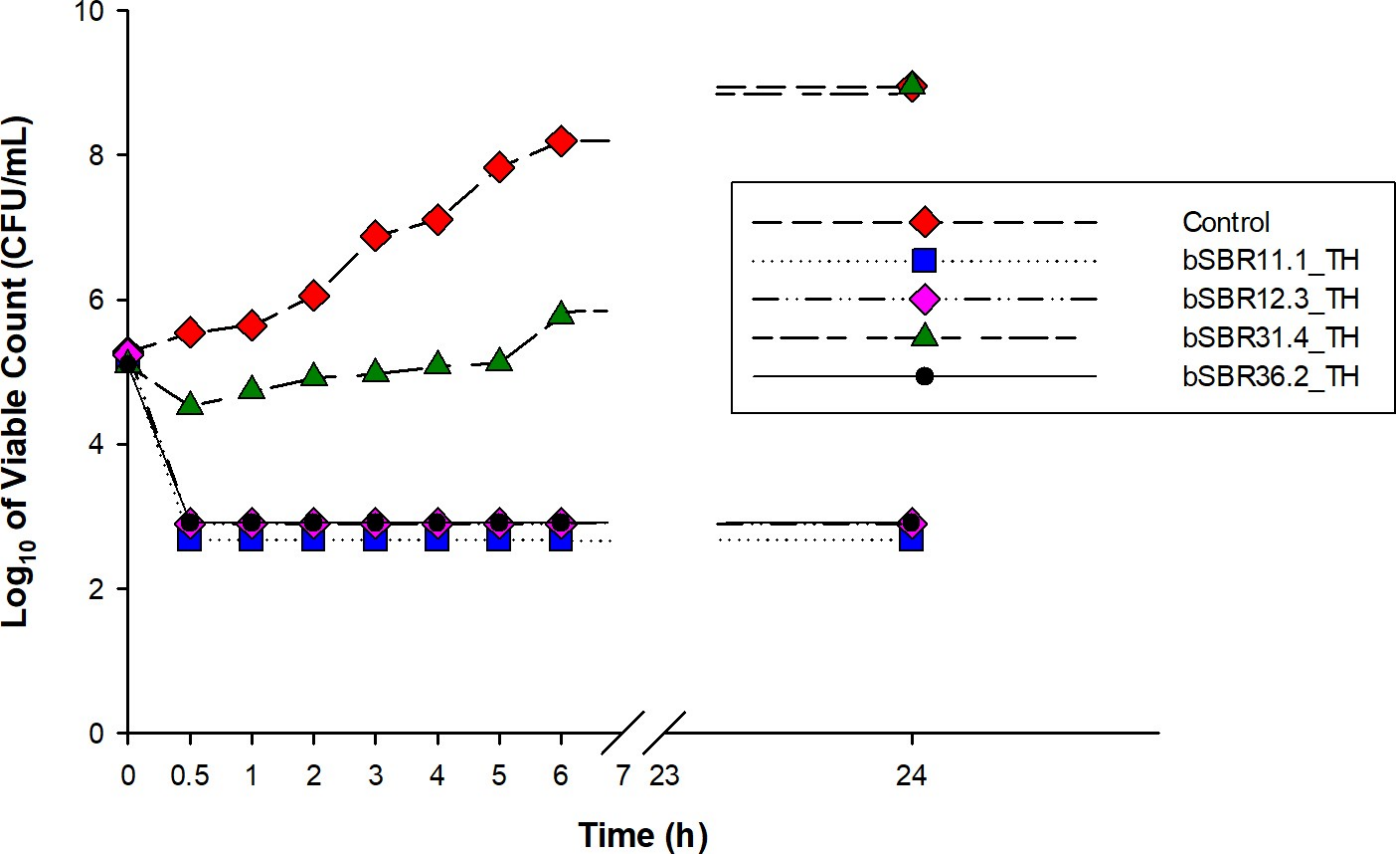
Time-kill curves for *S*. *aureus* strain PB36 (MRSA) using four extracts, including bSBR11.1_TH, bSBR12.3_TH, bSBR31.4_TH, and bSBR36.2_TH compared with *S*. *aureus* strain PB36 (MRSA) cultured alone.

**Fig 3** shows time-kill curves for bacterial extracts against *S*. *aureus* strain PB36 (MRSA). *Photorhabdus* extracts of strains bSBR11.1_TH, bSBR12.3_TH, and bSBR36.2_TH exhibited a steady reduction of *S*. *aureus* strain PB36 (MRSA) from 10^5^ CFU/ml to 10^3^ CFU/ml within 30 min and did not recover within 24 h. *Xenorhabdus* extract (bSBR31.4_TH), on the other hand, increased with the growth of *S*. *aureus* strain PB36 (MRSA) was observed over 6–24 h. The controls revealed no reduction in viable count and steady growth throughout 24h. The variabilities of *S*. *aureus* strain PB36 (MRSA) was significant difference compared with control at all tested times (*p < 0*.*05*). However, the variabilities of *S*. *aureus* strain PB36 (MRSA) after exposure to the extract of *Photorhabdus* strain bSBR31.4_TH at 24 h was no significant differences compared with control (*p > 0*.*05*).

## Discussion

In the present study, the EPNs of six *Steinernema surkhetense* and two *Heterorhabditis indica* were isolated from soil samples in Saraburi province, central Thailand, and were identified based on ITS and 28S rDNA sequences. Our finding that *S*. *surkhetense* and *H*. *indica* were common EPN species found in several provinces of Thailand is consistent with a previous study [16,17]. Fourteen isolates of symbiotic bacteria were identified as *X*. *stockiae* (six isolates), *P*. *luminescens* subsp. *akhurstii* (five isolates), *P*. *luminescens* subsp. *hainanensis* (two isolates), and *P*. *asymbiotica* subsp. *australis* (one isolate). Our previous study reported that these bacteria were commonly found in several provinces of Thailand, including Phetchabun, Kanchanaburi, Nakhon Ratchasima, Nakhon Nayok, Khon Kaen, and Suphanburi [16,17].

The different strains of *Xenorhabdus* and *Photorhabus* have been reported with variation in antibacterial activity. In the present study, four isolates of *Xenorhabdus* and *Photorhabdus* extracts showed efficacy against many antibiotic-resistant bacteria. *Photorhabdus luminescens* subsp. *akhurstii* (bSBR36.2_TH) extract showed the most inhibitory effect compared with the bacterial isolates tested. The previous study showed that *P*. *luminescens* could inhibit the growth of *B*. *subtilis*, *E*. *coli*, *S*. *pyogenes*, and *S*. *aureus* RN4220 (drug-resistant and clinical isolate) [10]. *Photorhabdus* can inhibit the growth of up to 32 species of fungi [9]. In addition, trans-cinnamic acid (TCA), produced by *Photorhabdus*, showed the inhibition of *Colletotrichum gloeosporioides*, *C*. *fragariae*, and *C*. *acutatum* at 10–100 μg/ml. This substance also inhibits the growth of *Fusicladium effusum*, which is the cause of Pecan scab [35]. In addition, our study demonstrated that the ethyl acetate extract of bMW27.4_TH *P*. *temperata* subsp. *temperata* could inhibit up to 10 strains of drug-resistant bacteria. All *Photorhabdus* extracts of Mae Wong national park could inhibit *S*. *aureus* ATCC20475, *S*. *aureus* strain PB36 (MRSA), and *S*. *aureus* strain PB57 (MRSA) [36]. *Xenorhabdus* produced xenocoumacin derivatives [37] and amicoumacin derivatives [38], were found to be potent antibiotics against *S*. *aureus* [8], while all the *Photorhabdus* spp. produced isopropylstilbene [39,40], which had multiple biological activities, including antibiotic activity against *S*. *aureus* and *E*. *coli* [24].

Based on the MIC and MBC, the ability to inhibit the growth of antibiotic-resistant bacteria varied on each isolate of *Xenorhabdus* and *Photorhabdus*. This may be due to either the ability of each symbiotic bacterium to produce effective metabolites or the susceptibility of antibiotic-resistant bacteria. The MIC of *Photorhabdus* extracts on *S*. *aureus* strain PB36 (MRSA) was found in 0.98 mg/ml in this study. In contrast, the *Stephania suberosa* Forman extract (SSE) against ampicillin-resistant *S*. *aureus* showed higher MIC with 4 mg/ml [34]. High MIC was also noted in the olive oil polyphenol extract [41], *Camellia sinensis*, and *Azadirachta indica* leaves extracts [42] against *S*. *aureus*. This indicates that *Photorhabdus* extracts are more effective than SSE, olive oil polyphenol extract, *Camellia sinensis*, and *Azadirachta indica* leaves extracts.

The combination of bacteria extracts and antibiotics (oxacillin and vancomycin) exhibited no synergistic activity against *S*. *aureus* strain PB36 (MRSA). These results were in contrast with a previous study suggesting that *Stephania suberosa* Forman extract demonstrates synergistic interaction with ampicillin against the clinical isolates of *S*. *aureus* [34]. Moreover, the combinations of *Cyperus rotundus* L. extract and ampicillin antibiotics showed a synergistic interaction against *S*. *aureus* [43].

In terms of time kill assay for *S*. *aureus* strain PB36 (MRSA), the extracts of bSBR11.1_TH, bSBR12.3_TH, and bSBR36.2_TH had stronger bactericidal activities than extracts of bSBR31.4_TH. This result correlates with the MIC and MBC assays. Moreover, the assay time for killing *S*. *aureus* strain PB36 (MRSA) was rapidly limited to 30 min. These results differ from the previous findings, wherein *Stephania suberosa* Forman extract plus ampicillin antibiotic exhibited synergistic activity against the ampicillin-resistant *S*. *aureus* [34]. Apart from this, the combinations of *Cyperus rotundus* L. extract and ampicillin antibiotics showed that the killing of ampicillin-resistant *S*. *aureus* cells was dramatically reduced by these combinations [43]. Although, mechanism by which the products from *Xenorhabdus* and *Photorhabdus* exhibited activity against antibiotic-resistant bacteria is not known. The results from previous studies based on known antibacterial compounds produced from *Xenorhabdus* and *Photorhabdus* suggest that the products may inhibit bacterial growth by Xenocoumacins [7], Fabclavines, 1-carbapen-2-em-3-carboxylic acid [10], 2-isopropyl-5-(3-phenyl-2-oxiranyl) benzene-1,3-diol [25]. Confirmation of the mechanism and identification of the specific target on bacterial cell are important for the novel drug discovery. In addition, cytotoxic testing of the novel compounds could be promoting the use of the new drug. At present, a few toxins identified from *Photorhabdus* bacteria showed cytotoxicity to mammalian cells [44–46]. This indicates that several concerns of drug recovery need to be seriously considered before use.

In summary, fourteen isolates of EPNs were obtained from a total of 230 soil samples, with 46 soil sites collected from Saraburi province, central Thailand. *Steinernema surkhetense* and *Heterorhabditis indica* were the common species found in soil samples. For symbiotic bacteria, *Xenorhabdus stockiae* (six isolates), *P*. *luminescens* subsp. *akhurstii* (five isolates), *P*. *luminescens* subsp. *hainanensis* (two isolates), and *P*. *asymbiotica* subsp. *australis* (one isolate) were also isolated from EPNs in the Saraburi province. Based on antibacterial activity, *Photorhabdus* spp. showed the potential to inhibit the growth of *S*. *aureus* strain PB36 (MRSA). This finding might be useful in further drug discovery from natural resources.
